# Evaluation of disposable filtration systems for harvesting high cell density fed batch processes

**DOI:** 10.1186/1753-6561-5-S8-P73

**Published:** 2011-11-22

**Authors:** Antje Pegel, Friedemann Übele, Sven Reiser, Dethardt Müller, Gregor Dudziak

**Affiliations:** 1 Rentschler Biotechnologie GmbH, 88471 Laupheim, Germany

**Keywords:** filtration, single-use, disposable, CHO, high cell density, fed batch

## Introduction

In the underlying study we evaluated different single-use filtration systems for cell separation and harvest clarification in 1,000 L scale. A screening of different depth filters was carried out with various single-use filters from Pall, Cuno (3M), Millipore and Sartorius Stedim. In total, we included 85 depth filtrations in the screening. Out of that, two single-use filtration systems were chosen and further tested in 200 L scale. Based on these results, a single-use filtration set-up for harvesting production scale fed batch processes was determined.

## Material and methods

High cell density fed batch cultivations of a monoclonal antibody (mAb) expressing Chinese Hamster Ovary (CHO) cell line were harvested by depth filtration and 0.2 µm filtration after 14 to 19 days at viabilities ranging from 40 to 95 %. For the screening in 10 L scale, single-use depth filters (23 to 26 cm^2^) with different separation ranges were used (Table 1). Subsequently, two disposable depth filtration systems were tested in 200 L scale using filter capsules with a filter area of 0.23 to 0.25 m^2^. Depth filtrates were 0.2 µm filtered with Pall EKV (20 cm^2^). During filtration, a constant flow of 100 L·m^-2^·h^-1^ was applied. Maximum capacities of the filters were determined at a pressure of 1 bar. Filter performance was assessed with regard to filter capacity, filtrate turbidity and product yield. Furthermore, content of DNA and Host Cell Protein (HCP) in filtrates were measured.

**Table 1:** Characteristics of the different single-use depth filters

Manufacturer	Material*	Filter type	Retention range (µm)*	Number of filter layers*
		PDK7	20 – 4	
		PDK6	20 – 3	
Pall Seitz^®^ HP-Series	Cellulose, Diatomaceous earth, Resin	PDK5	20 – 1	2
		PDH4	15 – 0.4	
		PDE2	3.5 – 0.2	
	
Seitz^®^ P-Series		KS50P	0.8 – 0.4	1

		10SP02A	7 – 1	
Cuno Zeta Plus^®^	Cellulose, Diatomaceous earth, Perlite	30SP02A	5 – 0.8	2
		60SP02A	5 – 0.65	
		60ZA05A	0.8 – 0.6	

Millipore Millistak+^®^	Cellulose, Diatomaceous earth	D0HC	9 – 0.6	2
		C0HC	2 – 0.2	

Sartorius Sartoclear P^®^	Cellulose, Diatomaceous earth, Binding matrix	PB1	11 – 4	2
		PB2	8 – 1	

* Data according to manufacturers [http://www.pall.com, http://www.cuno.com, http://www.millipore.com, http://www.sartorius-stedim.com].

## Results

### Depth filter screening

**Figure 1:**
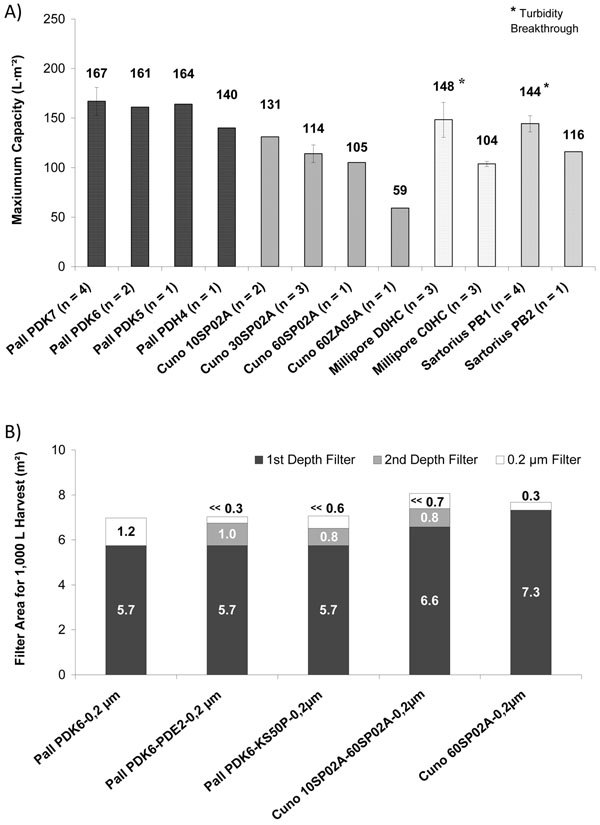
**A)** Maximum capacities of depth filters in small scale screening. **B)** Calculated filter areas for filtration of 1,000 L harvest based on results of large scale trials.

The filters PDK7, PDK6 and PDK5 from Pall showed the highest maximum capacities with 161-167 L/m^2^ (Figure 1 A). These double layered filters had identical first membranes and differing finer second membranes. Filtrate turbidity was below 7 NTU when applying PDK6, whereas in filtrates generated with the coarser filter PDK7 turbidities up to 9 NTU were observed. Additionally, product loss with PDK6 (6 %) was lower compared with PDK7 (8 %) or PDK5 (13 %). For that reason Pall PDK6 was selected for the scale-up experiments.

Additionally, the depth filters 10SP02A and 60SP02A from Cuno were chosen. For filter 10SP02A a maximum capacity of 131 L/m^2^ was obtained. However, the filtrate turbidity was higher than 10 NTU causing a fast blocking of the 0.2 µm filter. Therefore, this filter was combined with the finer depth filter 60SP02A. The filter 60SP02A was selected due to turbidity values below 10 NTU and an acceptable capacity of 105 L/m^2^ when applied stand-alone.

Turbidity breakthroughs at pressures below 1 bar were observed for the depth filters Millipore D0CH and Sartorius Stedim PB1 (Figure 1 A). Consequently, these filters were not considered for the scale-up studies.

### Scale-up

The selected depth filters were applied in 200 L scale using the Stax™ Disposable Depth Filter System (Pall) and the Zeta Plus™ Encapsulated System (Cuno), respectively. Performance of depth filters was comparable in large scale and small scale. Maximum capacities in the large scale trials were 174 L/m^2^ for Pall PDK6, 152 L/m^2^ for Cuno 10SP02A, and 137 L/m^2^ for Cuno 60SP02A. Product loss was below 10 %.

Based on maximum capacities filter areas were calculated for harvest in 1,000 L scale (Figure 1 B). An optimal filtration set-up leading to the lowest total filter area (6.9 m^2^) was found in the depth filter Pall PDK6 and a subsequent 0.2 µm filter. Insertion of a second depth filter after Pall PDK6 reduced the area of the 0.2 µm filter but did not affect the total filter area. The depth filter area of Cuno 60SP02A (7.3 m^2^) was comparable to that of the filter combination Cuno 10SP02A – 60SP02A (7.4 m^2^).

DNA content in the filtrate was reduced by 30 % with Pall PDK6 and even by 70 % with the additional depth filter PDE2. The Cuno depth filter combination 10SP02A – 60SP02A reduced DNA content by 99 % compared to 90 % when only applying 60SP02A. With the Pall depth filters a reduction of HCP by 30 % was measured whereas no HCP removal was observed for the Cuno depth filters.

## Conclusion

In this concept study disposable filtration systems were successfully tested in terms of identifying suitable filtration set-ups for equipping a 1,000 L disposable manufacturing line. These single-use filtration systems can thereby replace conventional (stainless steel) disc centrifuge and filtration steps in industrial mammalian cell culture production processes. The Stax™ system from Pall equipped with the filter PDK6 followed by a 0.2 µm filtration was identified as the first choice single-use filtration set-up offering high capacities and a low product loss. Addition of a finer second depth filter can further increase filtrate clarification resulting in a reduction of DNA and HCP in filtrates and a smaller area of the subsequent 0.2 µm filter, but is also combined with a higher risk of product loss and an increased time and handling effort.

